# Smile Train: The ascendancy of cleft care in India

**DOI:** 10.4103/0970-0358.57186

**Published:** 2009-10

**Authors:** Subodh Kumar Singh

**Affiliations:** G S Memorial Plastic Surgery Hospital, Varanasi, India

**Keywords:** Cleft lip, Cleft Palate, Out-reach programmes, Smile Pinki, Smile Train

## Abstract

Though India has an estimated population of one million untreated cleft patients, facilities for its treatment have been limited and are not evenly distributed across the country. Furthermore, a paucity of committed cleft surgeons in fewer hospitals to provide quality surgical treatment to these patients, poverty, illiteracy, superstitions and poor connectivity in some remote regions severely limit the chances of an average cleft lip patient born in India from receiving rational and effective comprehensive treatment for his/her malady. The Smile Train Project with its singular focus on cleft patients started its philanthropic activities in India in the year 2000. It made hospitals and included clefts surgeon equal partners in this programme and helped them treat as many cleft patients as they possibly could. The Project encouraged improvement of the training and infrastructure in various centres across the length and breadth of the region. The Project received an unprecedented success in terms of growth of number of centres, cleft surgeons and quantum of cleft patients reporting for treatment. The G S Memorial Hospital is one such partner hospital. It started innovative outreach programmes and took a holistic view of the needs of these patients and their families. With the support of the Smile Train, it has not only succeeded in providing treatment to more than 14,500 patients in 5 years, but has also devised innovative outreach programmes and seamlessly incorporated salient changes in the hospital system to suit the needs of the target population.

## INTRODUCTION

The Oscar winning documentary film ‘Smile Pinki’ highlighted the plight of the cleft lip patient in developing countries. The film stood testimony to the missionary passion of dedicated doctors, social workers and paramedical staff toiling in the Indian heartland as they treated hapless children afflicted with this unfortunate deformity with the support of the Smile Train Project.

Until a few years back, of the 35 000 cleft children born every year in India, only one-third received surgical correction; a mere half of these at the hands of a trained surgeon. A large majority of patients even today do not seek treatment and are resigned to their fate ascribing the condition to be a curse from God. This was compounded by the fact that treatment was lacking in rural areas and cities in some states of the country since government hospitals could not provide treatment for clefts. While treatment in private hospitals was too expensive for these patients, the few medical colleges proficient in cleft care failed to cope with an endless stream of patients. India, therefore, became a country with the unsavoury distinction of nursing the biggest backlog of unoperated cleft patients in the world.

These hapless children were condemned to a life of neglect and ridicule, and the total lack of self-esteem became their ill-fated destiny. The national government did not consider it as a deformity, and therefore there were no programmes promulgated for either the treatment or rehabilitation of cleft patients. In this environment, many Indian plastic surgeons, NGOs and itinerant through altruistic foreign missions provided free surgeries in camps. Complication rates were high, results were less than desirable and follow-up of cases done by these ‘parachute missions’ left much to be desired. Most of the times only lip was repaired and patients with cleft palate were asked to come later. Most of the surgeries were performed by residents without any supervision.

The advent of The Smile Train Project in India, coupled with the unwavering commitment of the Indian plastic surgeon, ushered in a new era in the history of management of cleft patients in South Asia. Today, more than 250 cleft surgeons in 160 partner hospitals operate upon over 50 000 patients annually; a five-fold increase in just 5 years! It is emphasized that in the last 9 years, more than 200 000 cleft patients received free treatment in Smile Train partner hospitals in India alone. All these hospitals and surgeons went through a thorough accreditation process to ensure safety and quality of care. These centres are now homogenously spread over almost all parts of India from Manipur in the East to Gujarat in the West, and Jammu and Kashmir in the North to Kanya Kumari in the South. Many cities have more than one centre. Varanasi, the birthplace of Sushruta, has the distinction of the highest rate of cleft surgeries in the world in its four centres. This is a tribute to the Father of Plastic Surgery.

## DIVERSE CHALLENGES IN INDIA

### The backlog

India has an estimated backlog of 1 000 000 cleft patients. A total of 35 000 new cleft patients are born every year. With the capacity to operate on approximately 50 000 patients each year, it is extrapolated that only 15 000 patients from the national backlog can be operated upon each year if our capability is not augmented. The frightful realization that it will take us about 100 years to clear our present backlog is disconcerting. As a corollary, a large number of patients will never benefit from modern medicine in their lifetime. However, if we could increase our capacity by twofold, the backlog shall be cleared in a decade.

### Awareness

Despite sustained efforts, it is surprising how patients could remain unaware of the facilities in the areas where centres have existed for several years. A large population of such patients is extremely poor. They are also illiterate and keep the company of uneducated people. They do not read newspapers or watch TV. They get interested only if they are fortunate enough to be informed of this facility by a trusted relative or friend who has witnessed the miracles of cleft care in her family, village or community.

### Loss of work

Most of such families have many small children to be looked after by the working mothers. In some cases a trip to the hospital takes more than 7 days from their village, during which time they miss out on their livelihood. Even a few days off work is not sustainable for the family. In this socio-economic milieu, even the informed cleft family can ill afford to seek care at the nearest centre.

## SMILE TRAIN PROGRAMME AT THE G S MEMORIAL PLASTIC SURGERY HOSPITAL

**Figure 1:**
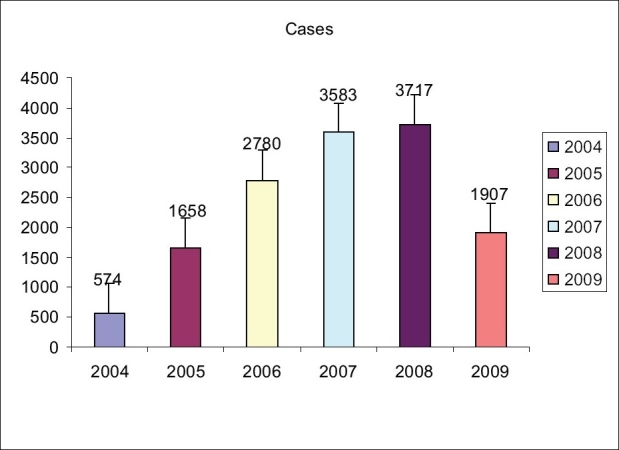
Yearly cases since the inception of programme in 2004

The Smile Train programme was commissioned at our hospital in April 2004 with a small team of doctors, paramedical and support staff. It was the 30th centre in India. With each passing year thereafter, the programme in this hospital went from strength to strength and from its humble beginnings a mere 5 years back, is today a success story. By the end of 2004, the hospital had provided treatment to 574 patients. In the subsequent year, the number tripled to 1658. The cleft programme run by the hospital became so popular that an endless train of hopeful patients converged from all directions from neighbouring states. This was a fruition of an effective and innovative outreach programme initiated by us. By 2006, we were operating the largest of cases in the world. A total of 2780 surgeries were performed in 2006, 3583 in 2007 and 3717 in 2008. An estimated 4000 patients shall receive treatment in 2009 [Figure 1].

We recognized the need to have a bigger team and training for more plastic surgeons interested in cleft surgery. Many plastic surgeons contributed significantly by joining this hospital for 6 months to over 1 year. A significantly large number of surgeries also necessitated creation of a safe peri-surgical milieu and adherence to strict protocols.

### Outreach programmes

It has been our unwavering philosophy that a programme that merely provides infrastructure and facility for cleft surgery but lacks other components towards holistic care and rehabilitation, is doomed to failure in the socio-economic realities of our region. It was, therefore, vital to reach out to the patients’ family to instill confidence and make them equal partners in care. We meticulously planned our out-reach programme for diverse geographical regions and created a motivated outreach team, with a trained social worker as its leader.

### Identification of target areas

We considered entire Uttar Pradesh, Bihar, Jharkhand, Chhattisgarh, Madhya Pradesh and West Bengal, the most populous regions in the world, as potential areas and identified areas that had direct road or rail link with Varanasi. Areas that had major plastic surgery centres were excluded from our primary list.

We always selected a hospital, nursing home or health centre of good repute in the district headquarters and having good connectivity being situated near to a bus station or railway station so that patients coming from outside could access our services easily. The Head of the hospital or a person assigned by him was made the local coordinator. The leader of the outreach team communicated with the local coordinator, reported to him and sought instructions from him.

### Awareness campaign for camps

To spread news and awareness about the camp, a team was despatched into the targeted area about 10- 12 days prior to the camp. This team worked with the local coordinator and moved with a local person as a guide. The team of 3-4 persons moved in a car or jeep into the interior areas of the district and also in many other districts around it. They played pre-recorded announcements through megaphones while negotiating by car through populous areas and sparsely populated areas alike. Relevant posters and hoardings were displayed and especially in small local markets, bus stands, schools, village block and health centres. The team also distributed handbills and pamphlets in buses, trains and also in the village and market areas.

### Domiciliary visits

If the team came to know about a cleft patient, they visited his/her house, talked to the parents and gave them all the information about this programme along with assistance in reaching the camp site.

### Newspaper advertisements

Information about the camp was also given in the local newspapers in the form of a small advertisement to appear within 10 days of the camp. The newspaper advertisements were very effective, and even if the parents of the patients were illiterate, somebody who read the advertisement went to the patient's house and informed them about the camp or brought them to the camp. Advertisements in newspapers, however, are becoming increasingly more expensive.

### TV and radio announcements

Sometimes the announcements were also made through local TV and radio. They were, however, less effective tools of propaganda.

### Camp

**Figure 2:**
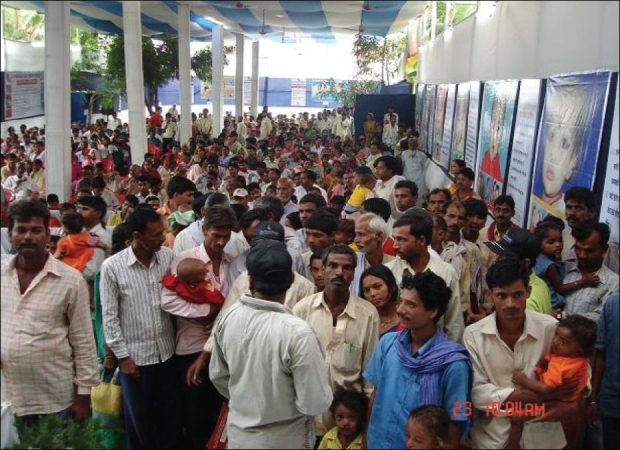
A typical cleft camp at G S Memorial Plastic Surgery Hospital

The camps were meant for evaluation and registration of the patients and surgery was never performed at the campsite. On the day of the camp, two plastic surgeons and two anaesthetists from our hospital would reach the campsite. The camp started at 0800 h. However, registration started very early in the morning and sometimes the evening before as many patients reach the campsite a night prior [Figure 2].

### Registration

Each patient is given two stickers to write their names and addresses (they are assisted). One sticker is pasted on the register and another on the patient's file. Each patient is examined by an anaesthetist and a plastic surgeon. They are advised haematinics and deworming medication, if needed. Then they are sent to the appointment desk where they are given a definite date for admission, taking into account their convenience and availability number of dates. They are also explained how to reach Varanasi and the Hospital; and the preparations required before coming to the hospital.

### Transport

Varanasi, being a holy city and a business centre, is well connected by rail and road network. It is strategically located in such a way that people can come to this city from neighbouring states by overnight train or bus service. As it is a small city, travelling in the city is not a problem. Most patients took a cycle ‘rickshaw’ or an ‘auto-rickshaw’ to reach the hospital.

### Hospital

It is a small private hospital and has 100 beds, mostly in semiprivate and general where attendants can stay with the patient. This is appreciated by the family of the Smile Train cleft patient.

### Special needs of cleft patients

Most of these patients are poor, illiterate and have never been to a city or a private hospital. Reaching the correct hospital is also a problem for them. They are easily recognized by the cleft in their lips and many middle-men and touts pester them to take them to other hospitals at the point of disembarkation itself. These patients are therefore handed over a hospital registration file before they commence the journey to the hospital.

### Creating systems that suit the needs of our target patients

Most of these patients are exceedingly poor, illiterate and have never used a modern toilet or bathroom. They have also never been to a city or a big building (hospital). They like to have self-cooked food. Only half of the patients come on a given date for surgery or follow-up. Although this was like a God sent opportunity for them, they still had priorities which were much more important for them- a job or work which was not available to them always. They took time to earn money or to borrow money to be able to come to the hospital and therefore missed their appointments. It was also difficult for the hospital staff to manage patients coming from such diverse conditions and areas. There is a great diversity in dialect and languages in this area. Most patients could not speak Hindi or English. They found it difficult to cope with the system of the hospital. Some were even taught how to use taps and toilet facilities in the hospital. So we realized the need to change the system of the hospital keeping in mind needs of our target patients. A continuous evaluation and feedback taken by our social workers helped us in this regard.

Patients were admitted for surgery or follow-up on all days and at all times irrespective of their dates of admission or follow-up. Their minor ailments like skin infections, upper respiratory tract infections, diarrhoea, etc were treated before surgery, which could take several days. They were kept in the hospital during this period. After surgery, patients were discharged from the hospital only after removal of stitches or after 5 days of palate surgery. It was difficult for patients to come back for stitch removal or to get treatment of any complications once they were back home. The average hospital stay was high for this reason but of great advantage to them. Patients insisted on staying in the hospital till they were absolutely well, even if it meant loss of earnings for them, as they were aware of poor medical support back home and the hospital always respected their desires and needs.

An area was designated for the attendants to cook their own food and and the hospital staff was always there to facilitate this. In hospital, social workers helped them at every step and also coordinated between them and hospital staff and kept patients well informed. Attendants were provided space-big halls to stay together, where they could meet each other and understand various things about hospital and treatment from one other.

### Admission and investigations

Most patients come with the camp registration file and are investigated and sent for further evaluation by the paediatrician. A thorough anthropometric evaluation is done for each patient. Anaesthetists and surgeons also evaluate each patient before surgery.

### Discharge

All the patients were discharged together on a particular day. Some common instructions were given to them by the hospital staff. They were also seen individually by the surgeon. This happy ending of their hospital stay was celebrated in a small function and all patients were given the Smile Train backpacks and some gifts and were encouraged to go to school. Needy patients also received money to meet their travel expenses.

### Smile train grant

The Smile Train also started a special grant for helping these patients with their conveyance expenses by providing clothes, toys, shoes, etc for the children and also for helping them with their educational needs, food, milk or shelter.

### Vocational training

A special pilot project was started in G S Memorial Hospital to provide some vocational training to the attendants of the patients while they were in hospital so that they could work on it and add to their family income. A good number of people took advantage of this programme.

### Average age at surgery

**Figure 3:**
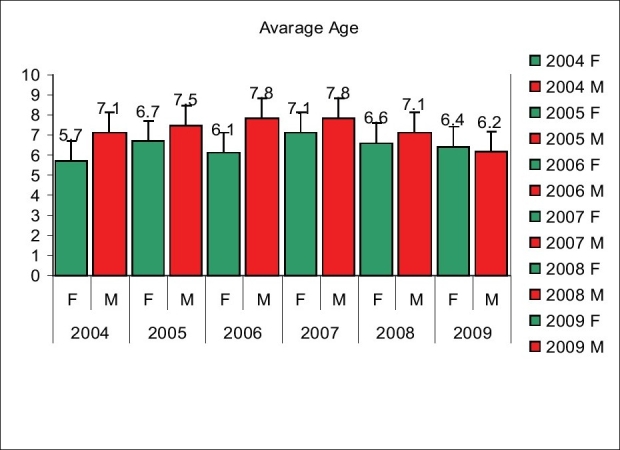
Average age of the patients at surgery

The average age of the patients at surgery may be a good indicator of backlog in that part of the country. Although in ideal situations the average age at surgery should have been below 1 year, the average age of patients in our hospital was 6.6 years in 2004. It slowly went up to 7.6 years in 2007 and then came down to 6.2 years in 2009 [Figure 3]. There were two reasons for it. First, the ongoing programme instilled confidence in people and even older patients who had settled with their deformities decided to take another chance for their surgery. Secondly, somewhere in 2006, the Smile Train decided to permit free surgeries to patients upto the age of 40 years.

**Figure 4:**
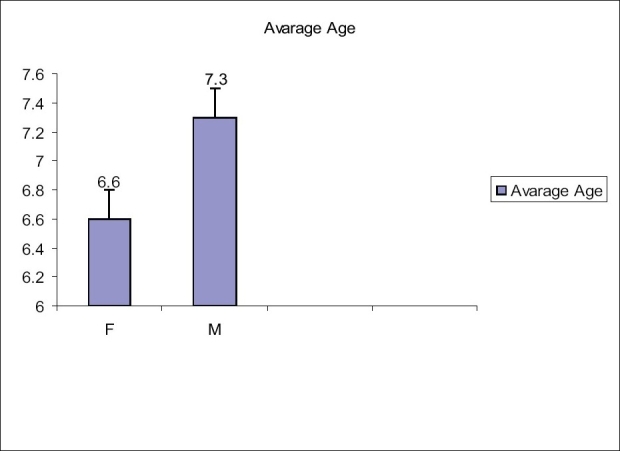
Average age at surgery for males and females

Interestingly, average age at surgery in girls was much lower (6.3 years) than that in boys (7.3 years). This was probably because of the greater concern of parents towards the girl child as she would more commonly be the target of ridicule and it will be difficult for parents to marry them to a suitable match [Figure 4].

### Record keeping

Photographs and clinical details of all the patients are meticulously maintained and are utilized for follow-up and research.

### Research

Even before starting this work with the Smile Train, we realized the tremendous potential of starting research to find the cause for clefting. We knew it requires a long research and we expected a big number of patients to be available. Our hospital initiated the genetic research along with the Human Genetics Department of BHU. The research involves evaluation of each patient, making of pedigree in familial cases, evaluation of maternal nutrition, biochemical analysis, extraction of DNA from the blood samples of patients, parents and relatives and their analyses by various methodologies and markers. The results so far have been very exciting and few papers have already been published and others are in the pipeline.[1–5]

## THE SMILE TRAIN AND ITS IMPACT IN INDIA

The Smile Train was started by Charles Wang and Brian Mullaney in 1999 in USA. It selected one congenital anomaly- clefts of the lip and palate and decided to do everything for it. The Smile Train did not believe in taking missionary surgeons to the place of need to operate upon cleft children. Instead, it believed in improving the infrastructure and training so that local hospitals and doctors could take care of their own patients. The Smile Train gives greatest emphasis on the safety and quality of surgery. It runs its programme through more than 1200 partner hospitals in 76 countries of the world where more than 2200 doctors treat cleft patients. More than 500 000 patients have been treated in these hospitals so far. The most successful Smile Train programmes run in India. Out of 500 000 patients, 200 000 have been treated in India (www.smiletrain.org).

Because of its policies and functioning, the Smile Train made a tremendous impact on the lives of cleft patients. This has become the greatest example in modern times, of how a single mission can transform lives of hundreds of thousands of patients suffering from one condition. This is also one of the finest examples of social entrepreneurship that works globally with highest efficiency and a minimum number of staff. This has been a ray of hope for those who never thought that happiness will ever come back to their lives. The number of cases that turned up for surgery surprised even most senior plastic surgeons. Registration in some of the camps organized by our hospital went up more than 500 in a day! Nobody believed that there were so many waiting patients for surgery. For the first time patients and parents had faith and hope that their defects can be fixed- free of charge. Suddenly, they wanted to live once again and they came up for surgery.

**Figure 5:**
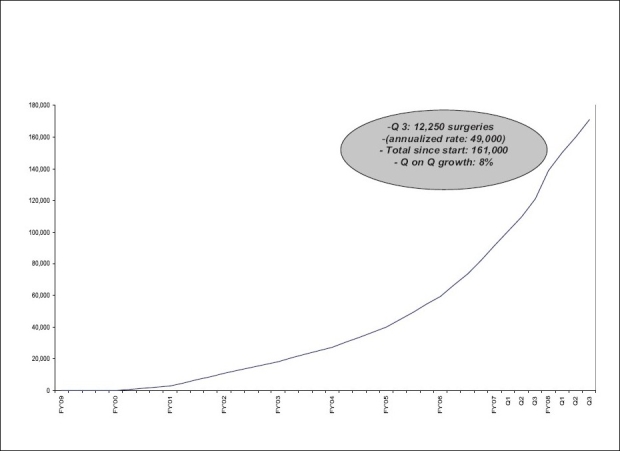
Growth of number of surgeries in Smile Train centres in India

The Smile train started functioning in India since the year 2000 with Mr. Satish Kalra as regional director (South Asia). The first few Smile Train centres were in southern India in Bangalore, Chennai and Thrissur. Soon many plastic surgeons came forward and expressed their willingness to work for this cause and became partners of the Smile Train [Figure 5]. By 2004, more than 30 centres started working in different parts of the country and this mission gathered momentum. Some of the centres in south, western and northern India were performing exceptionally well. They started organizing outreach programmes to reach out to these cleft patients in a very effective way. Patients had now developed faith in the Smile Train centres and they started coming to them in big numbers as the news spread by word of mouth.

**Figure 6:**
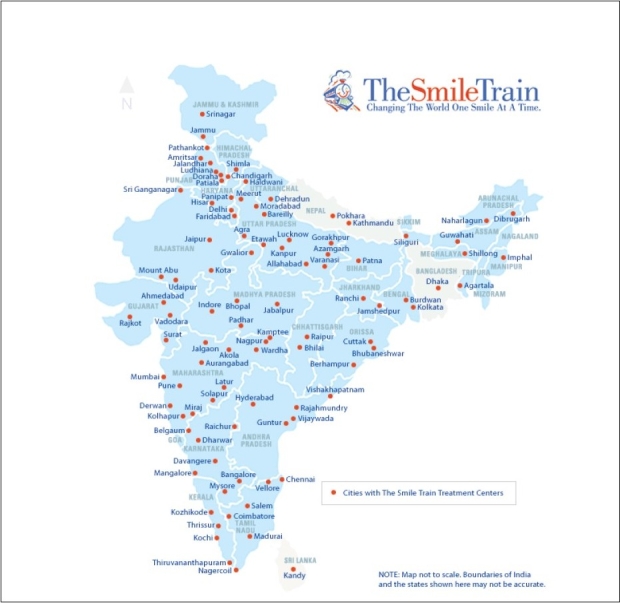
Smile Train treatment partner hospital locations in India

There was a steep rise in the number of centres following 2004 and more than 130 hospitals became its partners during this period. Similarly there was also a steep rise in the number of cleft cases being operated in these centres (Figures 5 and 6) [www.smiletrainindia.org]

The Smile Train did not only restrict itself to provide surgeries to the cleft children but also worked towards continuously improving the safety and quality parameters of hospitals as well as surgeons. It organized and supported academic activities related to the treatment of cleft and also contributed significantly to the development of speech therapy and orthodontics for cleft-affected children as a discipline and specialty.

**Figure 7:**
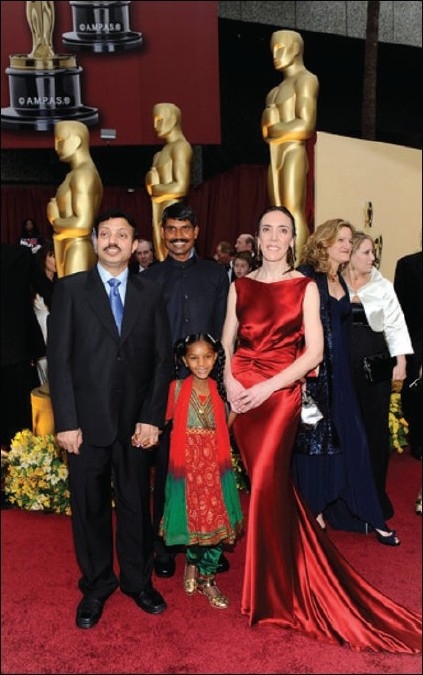
The photograph shows Pinki on the red carpet at the Academy Awards, Oscars flanked by her father Rajendra, his doctor, Dr. Subodh Kumar Singh and the film maker Ms. Megan Mylan

“Smile Pinki”, depicting the lives of two cleft children and the services being offered by a Smile Train partner hospital in India. Directed by Megan Mylan, a noted documentary film producer, it has since been screened at many film festivals and won world-wide acclaim when it won the 81^st^ Academy Award (The Oscar). This film was instrumental in bringing in tremendous amount of awareness about problems of cleft children in India and across the world [Figure 7].
